# Multiple sex chromosome system in penguins (*Pygoscelis*, Spheniscidae)

**DOI:** 10.3897/CompCytogen.v11i3.13795

**Published:** 2017-08-16

**Authors:** Ricardo José Gunski, Andrés Delgado Cañedo, Analía Del Valle Garnero, Mario Angel Ledesma, Nestor Coria, Diego Montalti, Tiago Marafiga Degrandi

**Affiliations:** 1 Universidade Federal do Pampa, São Gabriel, Rio Grande do Sul, Brazil; 2 Parque Ecológico El Puma- Posadas, Misiones, Argentina; 3 Depto. de Biología – Aves, Inst. Antártico Argentino, Buenos Aires, Argentina; 4 Universidade Federal do Paraná, Curitiba, Paraná, Brasil

**Keywords:** Sphenisciformes, karyotype, sex chromosomes, evolution

## Abstract

Penguins are classified in the order Sphenisciformes into a single family, Spheniscidae. The genus *Pygoscelis* Wagler, 1832, is composed of three species, *Pygoscelis antarcticus* Forster, 1781, *P. papua* Forster, 1781 and *P. adeliae* Hombron & Jacquinot, 1841. In this work, the objective was to describe and to compare the karyotypes of *Pygoscelis* penguins contributing genetic information to Sphenisciformes. The metaphases were obtained by lymphocyte culture, and the diploid number and the C-banding pattern were determined. *P. antarcticus* has 2n = 92, P. papua 2n = 94 and *P. adeliae* exhibited 2n = 96 in males and 2n = 95 in females. The difference of diploid number in *P. adeliae* was identified as a multiple sex chromosome system where males have Z1Z1Z2Z2 and females Z1Z2W. The C-banding showed the presence of a heterochromatic block in the long arm of W chromosome and Z2 was almost entirely heterochromatic. The probable origin of a multiple system in *P. adeliae* was a translocation involving the W chromosome and the chromosome ancestral to Z2. The comparison made possible the identification of a high karyotype homology in Sphenisciformes which can be seen in the conservation of macrochromosomes and in the Z chromosome. The karyotypic divergences in *Pygoscelis* are restricted to the number of microchromosomes and W, which proved to be highly variable in size and morphology. The data presented in this work corroborate molecular phylogenetic proposals, supporting the monophyletic origin of penguins and intraspecific relations.

## Introduction

In the class Aves, penguins are classified in the order Sphenisciformes in a single family Spheniscidae. The 18 extant species are divided in six genera, *Aptenodytes* Miller, 1778 (2 species), *Eudyptes* Vieillot, 1816 (6 species), *Pygoscelis* Wagler, 1832 (3 species), *Spheniscus* Brisson, 1760 (4 species), *Megadyptes* Milne-Edwards, 1880 (1 species) and *Eudyptula* Bonaparte, 1856 (2 species) (Stonehouse 1975). The monophyletic origin of penguins has been evidenced in different studies using morphological characters, DNA hybridization and more recently by molecular data with whole-genome analysis (Sibley and Ahlquist 1990, Giannini and Bertelli 2004, Baker et al. 2006, Jarvis et al. 2014).

Using mitochondrial and nuclear sequences, Baker and co-workers (2006) reconstructed the phylogeny of penguins and solved divergences observed in an intra genera relationship. The molecular dating estimated that ancestral penguins originated about 71 million years (Mya) ago in Gondwanaland while current species shows an Antarctic origin about 40 Mya. *Aptenodytes* (king and emperor penguins) was the first genus to diverge as basal lineage about 40 Mya. *Pygoscelis* branched about 38 Mya diversifying to *P. adeliae* Hombron & Jacquinot, 1841 in 19 Mya, *P. antarcticus* Forster, 1781 and *P. papua* Forster, 1781 at 14.1 Mya. The common ancestry of other genera was estimated at 27.8 Mya, followed by the division between genus *Spheniscus* and *Eudyptula* at approximately 25 Mya, followed by penguins *Megadyptes* and *Eudyptes*, about 15 Mya.

The karyotype information for Sphenisciformes is scarce, only five species have known diploid number. The black-footed penguin (*Spheniscus demersus* Linnaeus, 1758) has 2n = 72 (Jensen 1973), peruvian penguin (*S. humboldti* Meyen, 1834) has 2n = 78 (Takagi and Sasaki 1974) and magellanic penguin (*S. magellanicus* Forster, 1781) has 2n = 68 (Ledesma et al. 2003). Emperor penguin (*Aptenodytes forsteri* Gray, 1844) and king penguin (*A. patagonicus* Miller, 1778) have same diploid number 2n = 72 (Cardozo et al. 2003). Comparison of these karyotypes shows a numeric and morphological conservation of macrochromosomes among penguin species, indicating that differences observed in diploid number have their origin by fusion or fission involving in microchromosomes (Ledesma et al. 2003).

Related to sex chromosomes in Aves, it is known the chromosome system of type ZZ in males and ZW in females. The Z chromosome is relatively conserved among different orders, varying in size between the third and fourth pair of macrochromosomes (Takagi and Sasaki 1974), whereas the W chromosome presents significant differences, being of size similar to Z in Ratites to a small and heterochromatic chromosome in Passeriformes (Takagi and Sasaki 1974, Correia et al. 2009).

In this work, the goal was to describe the karyotype of *P. antarcticus*, *P. papua* and *P. adeliae*, contributing to the karyotypic knowledge about the order Sphenisciformes and to compare it with species already described.

## Material and Methods

### Location and sampling

The field work was carried out in the Potter peninsula (62°15'S, 58°40'W), King George Island (62°02'S, 58°21'W), South Shetland Island (60°18'S, 1°22'W). Blood samples were taken with heparin for the following species: *P. antarcticus* (8 males and5 females), *P. papua* (7 males and 5 females) and *P. adeliae* (8 males and 5 females).

### Blood cultures

Blood samples were used for lymphocyte culture, according to Moorhead et al. (1960). Briefly, cultures were prepared using approximately 1ml of peripheral blood in 10ml of RPMI 1640 culture medium, supplemented with 20% fetal bovine serum, 0.25ml penicillin/streptomycin and 0.2ml phytohemaglutinin. Cultures were incubated at 39°C for 72 hours. One hour before the incubation end, cells were treated with 0.1ml of colchicine solution (0.05%). After incubation, samples were treated with 10ml of 0.075M KCL for 20 minutes and fixed in a methanol and acetic acid (3:1) solution.

### Chromosomal analysis

For chromosomal analysis, the metaphases were stained with Giemsa solution. For each specimen, 40 metaphases were observed and photographed to assemble the karyotypes. Morphological classification of each chromosome pair was made according to Guerra (1986). CBG banding was conducted in sequential analysis, it was used slides preparations with a conventional staining and procedures of C-banding according to Sumner (1972).

## Results

**Figure 1.**
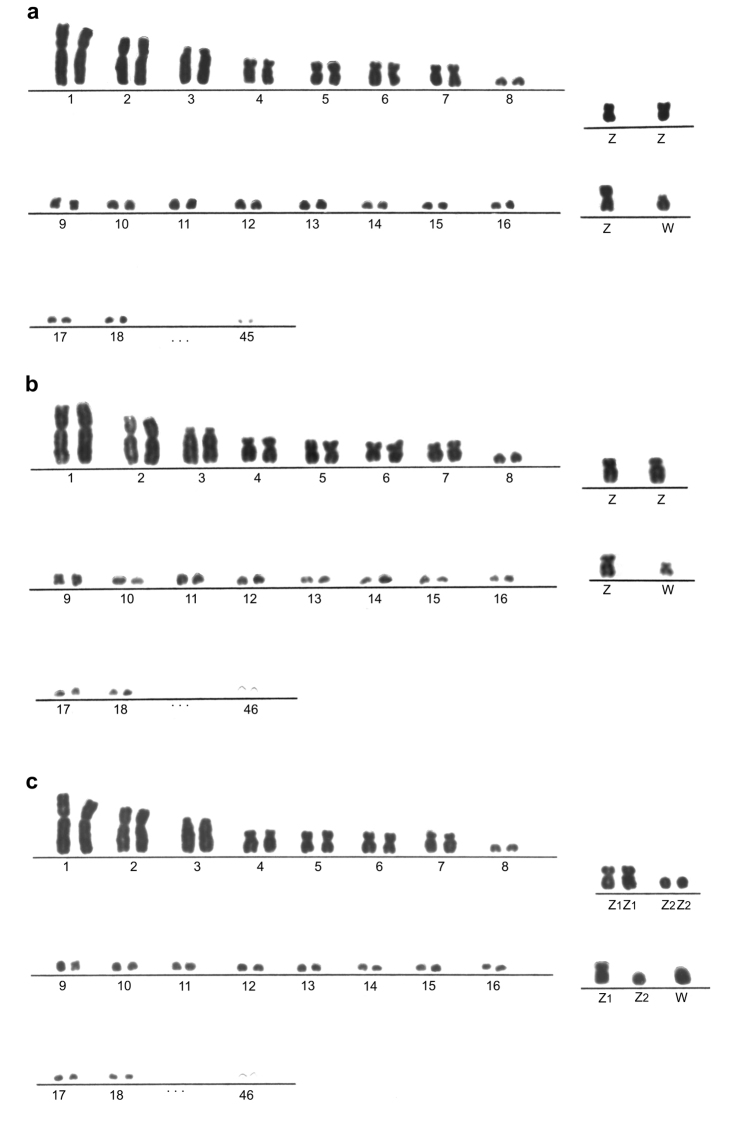
Partial karyotypes of *Pygoscelis* penguins **a** *P. antarcticus* 2n = 92 **b** *P. papua* 2n = 94 **c** *P. adeliae* 2n = 96 in males and 2n = 95 in females.

*Pygoscelis antarcticus* has 2n = 92 chromosomes (Fig. 1a). The karyotype is formed by seven pairs of macrochromosomes and the remaining were 38 pairs of microchromosomes. Sex chromosome Z was identified as submetacentric corresponding to the size of the 4^th^ pair and W is acrocentric corresponding to size of the 8^th^ pair.

*Pygoscelis papua* showed 2n = 94 (Fig. 1b). The karyotype exhibited the same morphological characteristic such as in *P. antarcticus*, with seven autosomal pairs of macrochromosomes and the divergence was observed in the number of microchromosomes, 39 pairs. The Z chromosome was submetacentric and W was metacentric.

For the *P. adeliae* it was observed that there is a difference in the diploid number between males and females. The males had 2n = 96, while the females showed 2n = 95 (Fig. 1c). The autosomal complement was formed by seven pairs of macrochromosomes highly similar at *P. antarcticus* and *P. papua*, the remaining were microchromosomes. The sex chromosomes in males were identified as one pair of submetacentric chromosomes (Z_1_Z_1_) and one small pair of telocentric denominated as Z_2_Z_2_. In females *P. adeliae* the sex chromosome system Z_1_ Z_2_W was identified, and the W chromosome was telocentric.

The classification of chromosome morphology in *Pygoscelis* confirmed the conservation of seven macrochromosomes and the Z chromosome between species. The pairs 1, 2, 4, 6, 7 and Z are submetacentric, pair 3 is acrocentric and pair 5 is metacentric. The W chromosome is acrocentric in *P. antarcticus*, metacentric in *P. papua* and telocentric in *P. adeliae* (Table 1).

**Figure 2.**
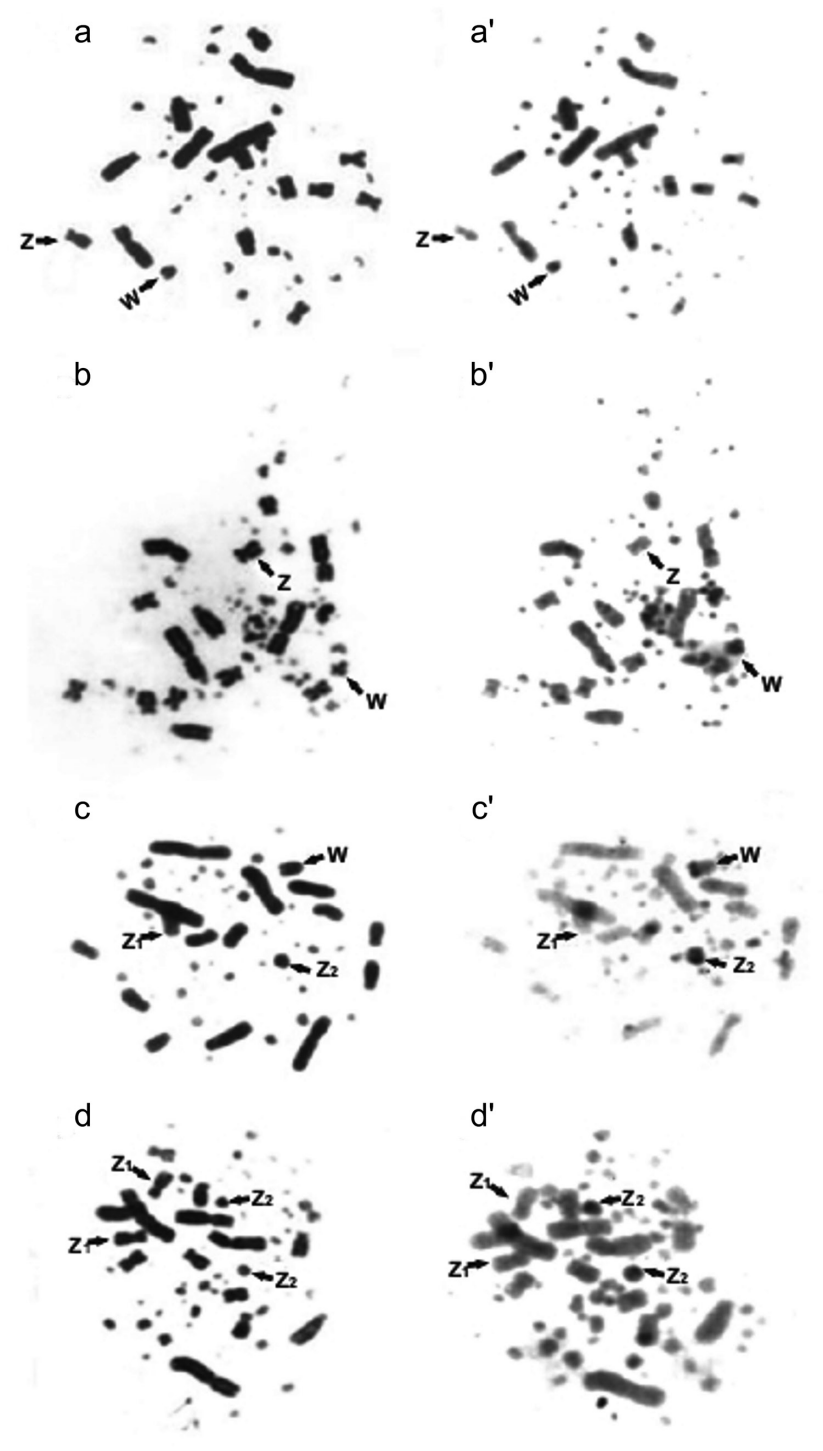
Metaphases in sequential Giemsa-C banding analysis **a-a**’ female of the *P. antarcticus* **b-b**' female of the *P. papua*, **c-c**' female and **d-d**' male of *P. adeliae*. The arrows show the sex chromosomes Z, Z_2_ and W.

The sequential Giemsa and C-banding analysis in *P. antarcticus* (Figs 2a–a') showed centromeric C-positive heterochromatin blocks in macro and microchromosomes; the Z chromosome was C negative. The W chromosome was slightly reactive to the banding. For the female *P. papua* (Figs 2b–b'), centromeric markings on macro and microchromosomes were observed. In the species *P. adeliae*, both sexes, female (Figs 2c–c') and male (Figs 2d–d') were analyzed. The macro and microchromosomes and the Z1 chromosome were little reactive and not showed marking in the centromere. The W chromosome has a characteristic heterochromatin block in the terminal region and the Z2 chromosome is completely heterochromatic.

**Table 1.** Comparison of the diploid number and morphology of the macrochromosomes and sex chromosomes of Sphenisciformes species.

Species	Common name	2n	1	2	3	4	5	6	7	Z	W	Z2	Reference
*Spheniscus demersus*	Black-footed	72	S	M	A	M	M	M	M	M	S	–	Jensen 1973
*Spheniscus magellanicus*	magellanic	68	S	S	A	S	M	S	S	S	S	–	Ledesma et al. 2003
*Spheniscus humboldti*	Peruvian	78	S	S	A	S	M	M	S	S	S	–	Takagi and Sasaki 1974
*Pygoscelis antarcticus*	chinstrap	92	S	S	A	S	M	S	S	S	A	–	This work
*Pygoscelis papua*	gentoo	94	S	S	A	S	M	S	S	S	M	–	This work
*Pygoscelis adeliae*	adelie	95–96	S	S	A	S	M	S	S	S	T	T	This work
*Aptenodytes forsteri*	emperor	72	S	S	A	S	S	M	M	S	*	–	Cardozo et al. 2003
*Aptenodytes patagonica*	king	72	S	S	A	S	M	S	S	S	S	–	Cardozo et al. 2003

M= Metacentric; S= Submetacentric; A= Acrocentric; T= Telocentric; - = absent; * Unanalyzed female.

## Discussion

*P. adeliae* (2n = 95–96), *P. papua* (2n = 94) and *P. antarcticus* (2n = 92) showed typical avian karyotypes. When compared to ancestral species such as Paleognathae (Nishida-Umehara et al. 2007) and Passeriformes (Kretschmer et al. 2014), it is remarkable that the chromosomal organization in macrochromosomes and microchromosomes has been maintained throughout evolution.

The diploid number of *Pygoscelis* is slightly elevated in relation to the values observed in more than 60% of the known Avian karyotypes, which correspond to 2n = 74–86 according to Rodionov (1997). Nevertheless, the conservation of macrochromosomes morphology suggests that karyotypes differences among these species are due to variations which occurred in microchromosomes. Considering the proposed phylogeny by Baker et al. (2006), in which *P. adeliae* (2n = 95–96) was the first species of the genus to diverge 19 Mya, it is suggested that a diploid number reduction occurred in *P. papua* (2n = 94) and *P. antarcticus* (2n = 92). This reduction can be attributed to chromosomal fusions involving microchromosomes.

The numerical and morphological conservation of the macrochromosomes in *Pygoscelis* is shared with *Spheniscus* and *Aptenodytes* species (Table 1). Ledesma et al. (2003) was the first to identify that, despite slight variations in the diploid number, Sphenisciformes showed high chromosomal homology. This homology extends not only to autosomal macrochromosomes, but also to the Z chromosome which is observed as submetacentric in seven of the eight species with known karyotypes. The small variations that can be observed in the morphology of the macrochromosomes correspond to a thin line dividing a chromosome to be classified as metacentric or submetacentric, such as in par 6 and 7 of the king and emperor penguins according to Cardozo et al. (2003). These homologies can be certainly confirmed by chromosome painting with fluorescent *in situ* hybridization experiments.

**Figure 3.**
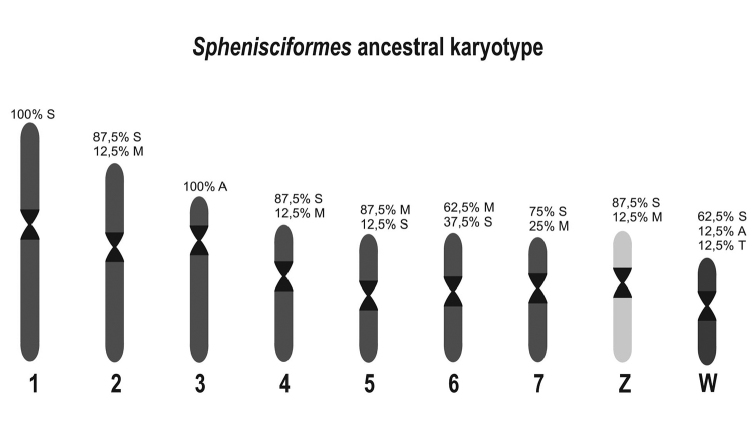
Hypothetical Sphenisciformes ancestral karyotype representing the conserved morphology of the macrochromosomes and ZW chromosomes. The frequencies of the morphologies (Metacentric= M; Submetacentric= S; Acrocentric= A; Telocentric= T) in the current species are represented above each pair.

Furthermore, on the basis of these observations, in this work we propose an ancestral karyotype for the present penguin species (Figure 3), based on the frequency of macrochromosomes and the sex chromosomes Z and W (Table 1). According to Baker (2006) penguins from the genus *Spheniscus* diverged from the *Eudyptula* penguins, thus it is quite probable that karyotypes highly similar are observed in *Eudyptula minor* Forster, 1781 and *E. albosignata* Finsch, 1874, whose karyotypes are unknown.

The W chromosome seems to play an important role in chromosomal evolution of Sphenisciformes. It presented morphological variations (Table 1) especially in the species from the genus *Pygoscelis* ranging from to acrocentric in *P. antarcticus*, to metacentric in *P. papua* and telocentric in *P. adeliae*. The analysis of C-banding made possible the differentiation of *P. antarcticus* and *P. papua* (Figure 2 a' and b') karyotypes in relation to *P. adeliae* (Figure 2 c'). However, it was not possible to differentiate the karyotypes of the first two species. In fact, the male karyotypes can only be recognized by their different diploid number, while in females the distinction is facilitated by the presence of the W chromosome that presented variations in morphology.

### Multiple sex chromosome system in Adelie penguin

The most interesting result of this work was seen in a multiple sex chromosome system in *P. adeliae*, where males have Z_1_Z_1_Z_2_Z_2_ and female has Z_1_Z_2_W. In birds this observation is unpublished, in higher animals multiple systems are rare exceptions and can be found in fishes, reptiles (some snakes), in some mammals Monotremes (platypus and echidna), in marsupials, in some Neotropical Primates, in eight species of bats, in two species of antelope (White 1977, Yonenaga et al. 1979, Tucker and Bickham 1986, Rens et al. 2007, Steinberg et al. 2014).

According to White (1973), multiple sex chromosome systems have arisen as a result of rearrangements involving sex chromosomes and autosomes, by centric fusion, reciprocal translocation, centric fission or tandem fusions. Cioffi et al. (2011) emphasizes the importance of repetitive sequences in multiple forms of sex chromosomes from fish, generating differences in morphology and size among them.

**Figure 4.**
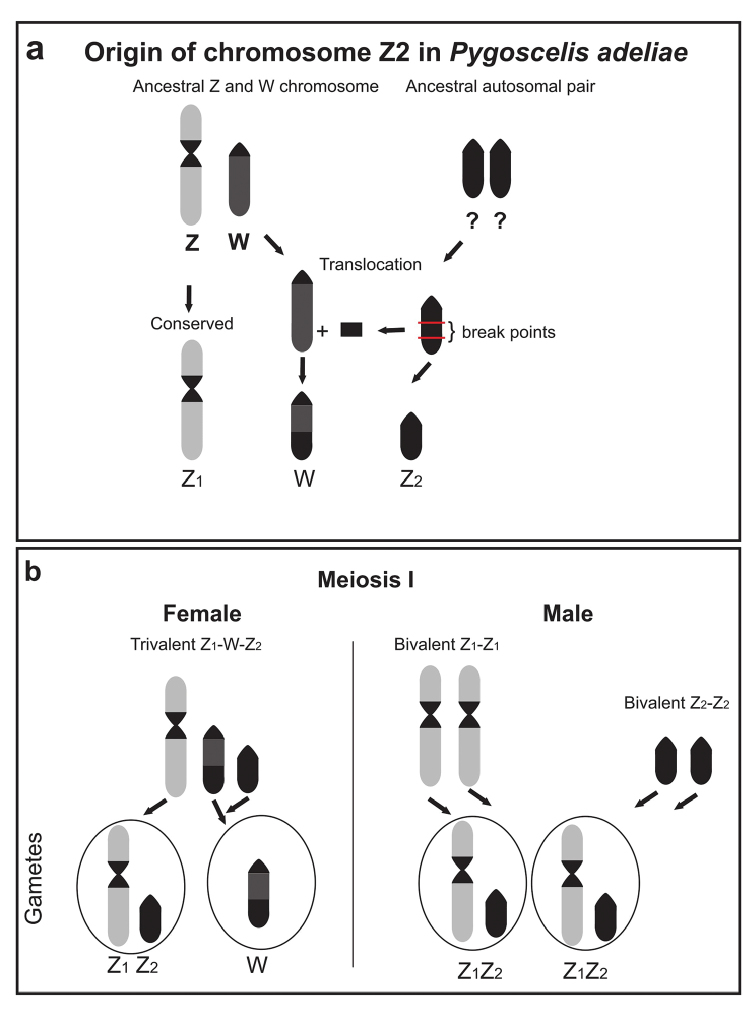
Schematic representation of the origin of the multiple sex chromosome system and a formation of the gametes during the meiosis I in *Pygoscelis adeliae* **a** The chromosomal translocation, which involving a heterochromatic segment of the ancestral chromosome Z2 with a terminal portion of the q arm of W chromosome **b** The meiosis in females and males of proposing the balanced gametes formation.

In the case of *P. adeliae* our hypothesis is that the multiple systems originated from a translocation involving the ancestral heterochromatic Z2 chromosome, with the terminal portion of the long arm of the W chromosome (Figure 4a). This hypothesis explains the presence of differentiated heterochromatic segment in the W chromosome (Fig. 2c'). In addition, the absence of this segment in the W chromosomes of *P. papua* and *P. antarcticus* suggests that this translocation occurred after their separation from *P. adeliae* or it was lost. A fairly similar heterochromatic labeling pattern was described by Toledo and Foresti (2001) on the X chromosome from the fish *Eigenmannia virescens* Valenciennes, 1842 and according to the authors these represent an early evolutionary stage of sex chromosome differentiation.

It is important to consider that the pairs Z2Z2 and Z1Z1 chromosomes, in males segregate normally during meiosis forming balanced gametes (Fig. 4b). In females, the formation of the trivalent Z1-W-Z2 must occur. The Z1 and Z2 segregate independently of the W chromosome. The W chromosome carries the translocated portion homologous to the Z2 chromosome (Fig. 4b). These hypotheses can be proven or refuted with the performance of meiotic analysis in females of *P. adeliae*.

In this work for the first time the karyotypes of *P. adeliae*, *P. papua* and *P. antarcticus* were presented. The presence of conserved macrochromosomes suggests that differences in diploid number 2n = 95-96, 94 and 92 are due to fusions between microchromosomes, reducing the diploid number. The results also point to a significant role of the W chromosome in speciation, with the first record of a multiple sex chromosome system in birds such as observed in *P. adeliae*. In addition, the comparison showed a high karyotype homology in Sphenisciformes which can be seen in the morphological conservation of macrochromosomes and in the chromosome Z.
